# Health Insurance Coverage and Unplanned Births in Rotterdam, the Netherlands: A Natural Experiment in the Generation R Study

**DOI:** 10.1111/psrh.70020

**Published:** 2025-06-21

**Authors:** Clair A. Enthoven, Jeremy A. Labrecque, Nicole Lucassen, Marco Varkevisser, Hilmar H. Bijma, Hanan El Marroun, Pauline W. Jansen

**Affiliations:** ^1^ Department of Child and Adolescent Psychiatry/Psychology Erasmus University Medical Center Rotterdam the Netherlands; ^2^ Department of Psychology, Education and Child Studies Erasmus School of Social and Behavioural Sciences, Erasmus University Rotterdam the Netherlands; ^3^ The Generation R Study Group Erasmus University Medical Center Rotterdam the Netherlands; ^4^ Department of Epidemiology Erasmus University Medical Center Rotterdam the Netherlands; ^5^ Department of Health Systems and Insurance Erasmus School of Health Policy & Management, Erasmus University Rotterdam the Netherlands; ^6^ Department of Obstetrics and Gynaecology, Division of Obstetrics and Fetal Medicine Erasmus MC Sophia Rotterdam the Netherlands; ^7^ Department of Care Ethics University of Humanistic Studies Utrecht the Netherlands

## Abstract

**Objectives:**

Starting January 1 2004, contraception was removed from the Dutch social health insurance for people aged 21 years and over. This study investigated the effect of social health insurance coverage for contraception on unplanned births.

**Methods:**

Data from the Generation R Study was used, a population‐based birth cohort of pregnant people with delivery dates between 2002 and 2006 (*N* = 2516) in Rotterdam, the Netherlands. Logistic regression models were constructed with a pre‐post policy variable, date of the last menstruation relative to January 12,004 and the interaction between them to allow the model to change over time with unplanned births as outcome, adjusted for age, migration background, educational level, household income and financial difficulties.

**Results:**

Removing contraception coverage from the social health insurance in 2004 showed a small increase in the odds ratio for unplanned birth, which was not statistically significant (OR = 1.18; 95% CI = 0.79–1.75). When participants with the last menstruation between January 1, 2004 and July 1, 2004 were excluded, a significant increase in the odds ratio for unplanned birth was found (OR = 2.69; 95% CI = 1.09–6.66).

**Conclusions:**

In our population of pregnant participants aged 21 years and older, we found tentative evidence that removal of contraception from the social health insurance may have led to a small increase in unplanned births with a time lag of 6 months. As this study only included pregnant people who gave birth, our results should be interpreted with caution and further research is needed for a definite conclusion on the effect of health insurance coverage on unplanned pregnancies.

**Abbreviation**
IUD: Intra Uterine Device

## 1 Introduction

In the Netherlands, family planning became increasingly well‐accepted after the introduction of oral contraception in 1962 [1]. Numbers on contraceptive use have traditionally been reported among women only, indicating that around 40% of the young women used the pill in 1968 and this percentage increased to 75% in 1974. An important contribution to this rise was the coverage by social health insurance (in Dutch: Ziekenfonds) in 1971 [1]. Since then, the use of contraception has been fairly stable, with 66% of the sexually active 15–49‐yearyear‐old women using any contraceptive method in 2017 [2]. As a result, the Netherlands became known for its relatively low rates of unwanted pregnancies and the low abortion rate [3]. However, more recent data showed that the use of contraceptive methods has decreased to 59% of the sexually active women between 15 and 49 years in 2023. Of those not using a contraceptive method, 2% reported the costs of financial burden [4]. Of course, it should be acknowledged that not only women may use contraception.

In the last decades, the reimbursement of modern contraceptive methods has been in and out of social health insurance coverage in the Netherlands. In 2004, contraception was removed from the social health insurance basic benefit package for those aged 21 years and over to reduce the increasing public spending on healthcare [5, 6, 7]. Currently (in 2025), most contraception methods (i.e., contraceptive pill, patch, ring, injection, implant, pessary, and intrauterine devices) are fully reimbursed from the mandatory private basic health insurance for those aged under 18 years. From age 18 to 21 years, contraception is subject to a mandatory deductible of €385, meaning that individuals must pay up to €385 out‐of‐pocket each year. This deductible can annually be voluntarily increased to €885 per year in return for a discount on their community‐rated basic health insurance premium [8]. From age 21 and over, contraception is only covered by additional health insurance.

Policy changes in healthcare may lead to changes in contraceptive use [9]. Much research has been performed on the impact of the Affordable Care Act (ACA) in the United States. The ACA had the aim to increase healthcare insurance coverage by broadening eligibility for Medicaid, an insurance program for the poor, as well as mandating the uninsured citizens to purchase, sometimes subsidized, private insurance [10]. Indeed, multiple studies confirmed an increase in service utilization, a decrease in out‐of‐pocket contraceptive costs, as well as changes towards more effective contraceptive methods, and potentially a lower risk of unintended pregnancy [9].

However, whether or not contraception should be reimbursed for everyone in the Netherlands is currently still under debate [11]. Opponents argue that there is no medical need to cover contraception in the basic health insurance package. Proponents argue that it is unfair that mostly women pay for family planning, that free contraception may increase contraceptive choices and satisfaction, and it may potentially also reduce the number of unplanned pregnancies [12]. However, empirical evidence for the latter claim is currently scarce. Therefore, the aim of this study is to investigate the effect of birth control coverage by mandatory social health insurance on the ratio of (un)planned births using data from a Dutch population‐based birth cohort of pregnant participants with delivery dates between 2002 and 2006.

## 2 Methods

### 2.1 Study Population

**FIGURE 1:**
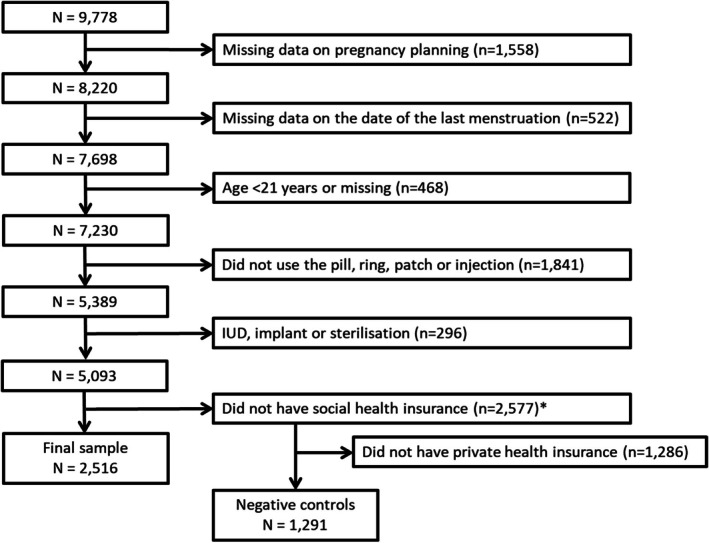
Flowchart of the Generation R Study population. IUD: intra uterine device. *The type of insurance for medical expenses was categorized into: Social health insurance (*n* = 2516; in Dutch *Ziekenfonds*), Civil service insurance (*n* = 318; e.g., IZA, IZR, and DGPV), private health insurance (*n* = 1291), no insurance (*n* = 5), or missing (*n* = 963).

The Generation R Study is a multi‐ethnic population‐based prospective cohort from fetal life onwards [13, 14]. Briefly, all pregnant people who intended to continue the pregnancy and who resided in Rotterdam with a planned delivery date between April 2002 and January 2006 (last menstruation dates between August 2001 and April 2005) were invited to participate by the Generation R research team. Of them, 9778 (participation rate 61%) enrolled. To study the trends in planned/unplanned births, we excluded from analyses: (1) participants with missing information on pregnancy planning (*n* = 1558); (2) participants with missing information on the date of the first day of the last menstruation (*n* = 522); (3) participants with missing information on age or who were younger than 21 years (*n* = 468), because contraception coverage was always available for them; (4) participants who did not use a contraceptive method that was affected by the policy change (pill, ring, patch, or injection) in the 5 years prior to pregnancy (*n* = 1841); (5) participants who reported sterilization or using an IUD or implant in the 5 years prior to pregnancy because these methods do not need to be replaced or only every three to ten years (*n* = 296); and (6) participants with civil, private or no insurance, because the policy likely only affected coverage of the social health insurance (*n* = 2577). The final study sample consisted of 2516 participants. Participants with private insurance were not affected because there was no uniform benefits package for them. We therefore used this sample as a negative control population (*n* = 1291) [15]. Figure 1 provides a schematic overview of the samples. The Medical Ethics Committee of Erasmus MC in Rotterdam, the Netherlands, has approved the study in accordance with the Declaration of Helsinki of the World Medical Association. Written informed consent was obtained from all participants.

### 2.2 Unplanned Pregnancy and Contraceptive Use

We measured pregnancy planning using a self‐report questionnaire at enrolment using a single question asking whether the pregnancy was planned or not. Since the cohort only included participants who decided to continue the pregnancy, we defined the ratio unplanned births versus planned births. We assessed contraceptive use using the question “Have you used contraceptives in the past 5 years?” If yes, follow‐up questions assessed which type they had used, with the categories contraceptive pill, intrauterine device, condom and/or other, such as contraceptive injection, ring, implant and/or patch (fertility awareness and withdrawal methods were not included). We allowed multiple answers.

### 2.3 Natural Experiment

We assessed the association between mandatory social health insurance coverage and unplanned pregnancy using a natural quasi‐experimental design [16]. The pre‐policy group consisted of participants with the last menstruation before January 12,004. The post‐policy group included participants with the last menstruation on or after this date. As the control and intervention group must be similar, we assessed multiple sociodemographic variables related to unplanned birth [17]. Age at conception was calculated as maternal age at birth minus gestational age at birth and categorized into 21–25, 26–30, 31–35 and > 35 years. Migration background was defined according to the classification of Statistics Netherlands. It was considered “yes” if one or both parents were born in another country than the Netherlands. Relationship status and educational level were assessed by questionnaire in the 12th week of gestation. Relationship status was categorized into married, cohabiting and single. Highest attained educational level was categorized into: Low (primary school; lower vocational training; intermediate general school; 3 years general secondary school), which typically corresponds to ≤ 12 years of education; Mid‐low (> 3 years general secondary school; intermediate vocational training; 1st year higher vocational training), in general corresponding with 13–15 years of education; Mid‐high (higher vocational training; Bachelor's degree), typically matching with 16 or 17 years of education; and High (higher academic education; PhD), usually indicating 18 years of education or more. Net household income and financial difficulties were self‐reported at the 30th week of gestation. Net household income was categorized as: Less than €1200/month; Between €1200 and €2000/month; and More than €2000/month, indicating the social security level and modal income in 2004. Difficulty in paying food, rent, electricity bill and suchlike was categorized into: No difficulty; Some difficulty; and Great difficulty.

### 2.4 Statistical Analyses

Prior to analyses, we handled missing values in sociodemographic factors using multivariate imputation by chained equations [18]. Besides the studied variables, we used pre‐pregnancy body mass index, parity, folic acid intake, number of sexual partners in the year prior to pregnancy, lifetime psychopathology (depression, anxiety, or eating disorder), and history of abortion as predictors for imputation [19].

We assessed the association between social health insurance coverage and unplanned pregnancies using logistic regression analyses. The pre‐post policy variable, defined by last menstruation before January 1, 2004 (coded as 0) or after January 1, 2004 (coded as 1) was the main determinant. To minimize bias from unmeasured macro‐level confounders (e.g., improved sexual education), we added a variable indicating the date of the last menstruation relative to January 1, 2004 in weeks to adjust for a general increase or decrease in unplanned births over time. An interaction term between the two variables was added to allow this general increase or decrease in unplanned births over time to be different and change discontinuously on January 1, 2004 before and after the policy change. This enabled us to investigate how much the odds of planned versus unplanned births changed right after the policy change.

To ensure identifying the true association of health insurance coverage, the pre‐policy and post‐policy groups should be very similar. After testing the differences before and after the policy change in the socioeconomic variables (Table 2) as well as visually inspecting the changes over time (Figure S1A–F), we adjusted for age, migration background, educational level, household income, and financial difficulties. All analyses rely on the assumptions that participants did not stock a large amount of contraception before the policy change or change the frequency of sexual intercourse because of the policy change and that no other policy that could affect the use of contraception and/or unplanned pregnancy was implemented at the same time. In two post hoc analyses, we first excluded participants with the last menstruation between January 1, 2004 and July 1, 2004 to reduce the possibility of still using oral contraception pills that were obtained before the policy change, and we secondly excluded participants who additionally reported using condoms or other barrier methods because these methods were not impacted by the policy change.

We used triangulation by repeating all analyses using propensity score matching. This way, the estimates of both modeling approaches can be compared to evaluate our confidence in the findings [20]. First, the propensity scores, which are the predicted probabilities of being in the pre‐policy or post‐policy group, were calculated given the covariates age, migration background, educational level, household income, and financial difficulties. Then, the participants were matched based on the propensity score using a 1:1 nearest neighbor matching with caliper width at 0.05 of the MatchThem package in R [21]. We repeated the analyses using propensity score matching among participants with some or great financial difficulties and among participants aged 21–25 years because the policy change may have had a greater impact on those groups.

Finally, we repeated the primary regression models, propensity score matching analyses, and potential significant effects from the post hoc analyses among participants with private insurance. Since contraception coverage did not change for this group on January 1, 2004, they served as a negative control population, for which we hypothesized no change in the ratio of planned versus unplanned births.

## 3 Results

The study population was on average 28.9 years (SD = 4.4) old at the last menstruation, 12.2% of them were single and around half (47.4%) had a migration background (Table 1). Twenty‐six percent of the participants had an unplanned birth.

**TABLE 1:** General characteristics of the total study population, and the study population before and after the policy change.

Variables	Total (*n* = 2516)	Before policy change (*n* = 1656)	After policy change (*n* = 860)	Difference
	Mean (SD) or N (%)	Mean (SD) or *N* (%)	Mean (SD) or *N* (%)	*P* *
Pregnancy planning				0.52
Planned	1851 (73.6%)	1225 (74.0%)	626 (72.8%)	
Unplanned	665 (26.4%)	431 (26.0%)	234 (27.2%)	
Age				0.65
21–25 years	704 (28.0%)	477 (28.8%)	227 (26.4%)	
26–30 years	989 (39.3%)	643 (38.8%)	346 (40.2%)	
31–35 years	692 (27.5%)	450 (27.2%)	242 (28.1%)	
> 35 years	131 (5.2%)	86 (5.2%)	45 (5.2%)	
Marital status				0.18
Married	1136 (46.0%)	734 (44.9%)	402 (48.2%)	
Cohabiting	1031 (41.8%)	689 (42.2%)	342 (41.0%)	
Single	300 (12.2%)	210 (12.9%)	90 (10.8%)	
N missing	49	23	26	
Migration background				< 0.01
Yes	1182 (47.4%)	747 (45.4%)	435 (51.2%)	
No	1311 (52.6%)	897 (54.6%)	414 (48.8%)	
N missing	23	12	11	
Educational level				< 0.01
Low	685 (28.0%)	460 (28.5%)	225 (27.2%)	
Mid‐low	1022 (41.8%)	687 (42.5%)	335 (40.5%)	
Mid‐high	458 (18.7%)	273 (16.9%)	185 (22.3%)	
High	279 (11.4%)	196 (12.1%)	83 (10.0%)	
*N* missing	72	40	32	
Household income				0.50
< €1200/month	471 (20.2%)	299 (19.6%)	172 (21.3%)	
€1200–€2000/month	557 (23.9%)	360 (23.6%)	197 (24.4%)	
> €2000/month	1303 (55.9%)	864 (56.7%)	439 (54.3%)	
*N* missing	185	133	52	
Financial difficulties				< 0.01
No	1831 (75.2%)	1244 (77.8%)	587 (70.1%)	
Some	508 (20.9%)	289 (18.1%)	219 (26.2%)	
Great	96 (3.9%)	65 (4.1%)	31 (3.7%)	
*N* missing	81	58	23	

* *p* values were calculated with chi‐square tests.

**FIGURE 2:**
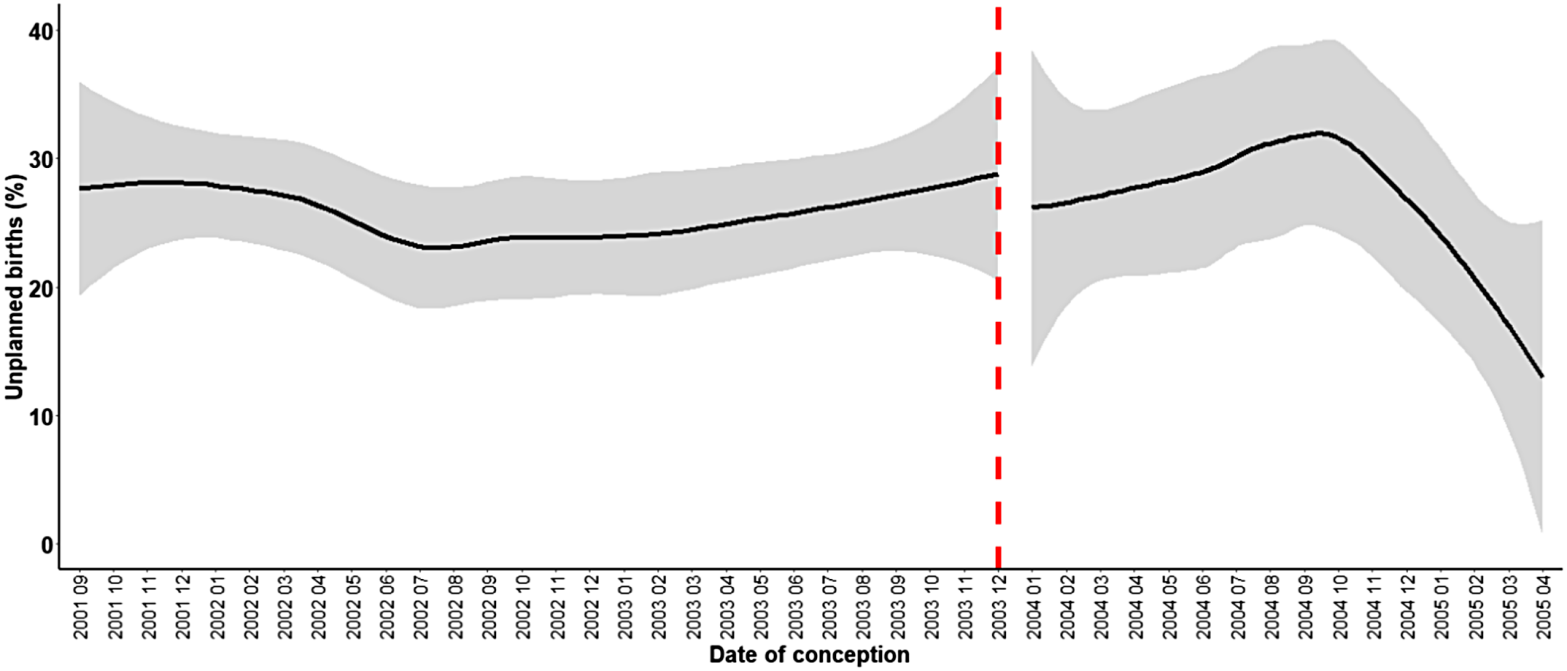
Percentage of unplanned births on the *y*‐axis and date of conception on the *x*‐axis, plotted using the local regression method. The black line indicates the percentage of unplanned births out of all births, the gray area indicates the standard error and the red dashed line indicates the timing of the policy change.

Removing contraception from the social health insurance in 2004 showed an increase in the odds ratio of unplanned versus planned births in the crude model (OR: 1.26; 95% CI: 0.87–1.82) as well as in the model adjusted for age, migration background, educational level, household income, and financial difficulties (OR: 1.18; 95% CI: 0.79–1.75), but both odds ratios were not statistically significant (Table 2). There was no significant change in unplanned versus planned births over time in the crude model (OR: 0.99; 95% CI: 0.92–1.05) and the adjusted model (OR: 0.99; 95% CI: 0.96–1.00; Table 2). The decrease over time was not significantly different before and after the policy change, as indicated by the non‐significant interaction terms in both models (*p* = 0.57 in the crude and *p* = 0.92 in the adjusted model; Table 2; Figure 2).

**TABLE 2:** Odd ratios for planned versus unplanned births in the unadjusted model (model 1) and the adjusted model (model 2).

*N* = 2516	Model 1		Model 2	
Predictors	OR	95% CI	OR	95% CI
Pre‐post policy	1.26	0.87; 1.82	1.18	0.79; 1.75
Time relative to 1‐1‐2004 in weeks	0.99	0.97; 1.02	0.99	0.96; 1.02
Pre‐post policy * Time relative to 1‐1‐2004	0.98	0.92; 1.05	1.00	0.93; 1.07
Age 26–30 years			0.66*	0.52; 0.83
Age 31–35 years			0.60*	0.46; 0.77
Age > 35 years			0.68	0.43; 1.07
No migration background			0.67*	0.55; 0.83
Mid‐low educational level			1.01	0.80; 1.27
Mid‐high educational level			0.73	0.53; 1.01
High educational level			0.57*	0.37; 0.86
Medium household income			0.65*	0.50; 0.85
High household income			0.45*	0.34; 0.60
Some financial difficulties			1.56*	1.23; 1.99
Great financial difficulties			2.04*	1.31; 3.18

* Significant association (*p* value < 0.05).

Post hoc analyses excluding participants with their last menstruation between January 1, 2004 and July 1, 2004 (total *N* = 2129) showed a significant increase in unplanned versus planned births after the policy change (OR = 2.69; 95% CI = 1.09–6.66; Table 3). Post hoc analyses excluding participants who additionally used condoms or other barrier methods in the previous 5 years (total *N* = 1819) showed no significant increase in unplanned births after the policy change (OR = 1.03; 95% CI = 0.63–1.68; Table 3).

**TABLE 3:** Odd Ratios for planned versus unplanned births, excluding participants with their last menstruation between January 1st 2004 and July 1st 2004 (model 3), and excluding participants who additionally used condoms or other barrier methods in the previous five years (model 4).

Predictors	Model 3	*N* = 2129	Model 4	*N* = 1819
	OR	95% CI	OR	95% CI
Pre‐post policy	2.69*	1.09; 6.66	1.03	0.63; 1.68
Time relative to 1‐1‐2004 in weeks	0.99	0.96; 1.02	0.98	0.95; 1.02
Pre‐post policy * Time relative to 1‐1‐2004	0.88	0.75; 1.02	1.03	0.95; 1.13
Age 26–30 years	0.70*	0.54; 0.90	0.72*	0.54; 0.94
Age 31–35 years	0.60*	0.45; 0.80	0.70*	0.51; 0.96
Age > 35 years	0.62	0.38; 1.03	0.91	0.54; 1.53
No migration background	0.68*	0.54; 0.85	0.62*	0.48; 0.80
Mid‐low educational level	0.97	0.76; 1.24	0.94	0.72; 1.23
Mid‐high educational level	0.66*	0.46; 0.94	0.57*	0.38; 0.86
High educational level	0.53*	0.34; 0.84	0.50*	0.28; 0.88
Medium household income	0.72*	0.54; 0.97	0.69*	0.50; 0.96
High household income	0.48*	0.35; 0.66	0.52*	0.37; 0.73
Some financial difficulties	1.58*	1.21; 2.05	1.61*	1.21; 2.14
Great financial difficulties	2.47*	1.52; 4.01	2.08*	1.24; 3.50

* Significant association (*p* value < 0.05).

Triangulation using propensity score matching showed similar results as the main analyses and post hoc analyses (Tables S1–S3). Subgroup analyses using propensity score matching among participants with some or great financial difficulties or a low household income (total matched *N* = 600) and among participants aged 21–25 years (total matched *N* = 440) showed no significant associations (respectively OR = 1.15; 95% CI = 0.55–2.29 and OR = 1.41; 95% CI = 0.62–3.23; Tables S4 and S5).

In the negative control population (*N* = 1291), a decrease in the odds ratio of unplanned versus planned births after January 1, 2004 was observed in the crude model (OR: 0.73; 95% CI: 0.35–1.56; Table S6), the model adjusted for age, migration background, educational level, household income and financial difficulties (OR: 0.70; 95% CI: 0.31–1.57; Table S7), and the propensity score matching analyses (OR: 0.83; 95% CI: 0.34–2.07; *N* = 916; Table S8), but all odds ratios were not statistically significant. After excluding participants with a last menstruation between January 1, 2004 and July 1, 2004, an increase in the odds ratio of unplanned versus planned births was observed in the adjusted model (OR: 1.80; 95% CI: 0.35–9.25; *N* = 1123; Table S9), and the propensity score matching analyses (OR: 2.24; 95% CI: 0.37–13.38; *N* = 582; Table S9), but both odds ratios were not statistically significant.

## 4 Discussion

In our cohort of pregnant participants aged 21 years and older in Rotterdam, The Netherlands, removing contraception from the social health insurance in 2004 did not significantly increase the rate of unplanned births right after the policy change in our total study population, nor in subgroups of participants with less financial means or participants aged ≤ 25 years. The policy change was, however, significantly associated with unplanned births when participants who became pregnant within the first 6 months after the policy change were excluded from analysis, potentially indicating that the impact of the policy change became apparent with a time lag of 6 months. This may be due to some participants, anticipating the policy change, arranging prescription or long‐acting contraception right before January 2004.

The main results of our study showed that the removal of contraception from the social health insurance basic benefit package did not significantly increase the rate of unplanned births among participants aged 21 and older. However, this should not be interpreted as evidence that any effect was absent. Though non‐significant, the effect estimates and confidence intervals of the crude model, the adjusted model, and the matched analyses are indicative of a direction towards an increase, which was absent in the analyses of the natural control population. In addition, post hoc analysis excluding participants who became pregnant in the first 6 months after the policy change showed a significant increase in unplanned births in the adjusted and the matched analyses. Although an increase was also observed in the natural control population, this was not significant with very wide confidence intervals. Since oral contraceptives are typically obtained for a six‐month period, it is perhaps not surprising that the effects of the policy change are not immediately observed, but rather occurred with a time lag when participants ran out of pills.

A few other studies focused on policy changes in financial coverage of contraception. Most of them studied contraceptive use after they were provided free of charge. For example, publicly funded oral contraception led to an increase in its use and a decrease in unplanned pregnancies in teenagers in Sweden [22], and a decrease in teenage pregnancies, induced abortions and repeat induced abortions in the United States [23]. In addition, an increased number of contraception prescriptions were observed after expansion of Medicaid coverage in the United States [24]. Reversely, a large increase in the costs of contraception resulted in more births among young mothers in Chili [25]. These results suggest that an increase in contraception costs may impact both the rate of induced abortion as well as unintended pregnancy among teenagers. This may explain the non‐significant findings in our study population consisting of participants who intended to give birth aged 21 years and older (because contraception has always been included in the basic benefit package for people below 21 years in the Netherlands).

This study had several strengths. First, the high response rate (61%) of pregnant people in Rotterdam who participated in the Generation R study resulted in a large sample size. Second, the novel design of the natural experiment allowed us to estimate the association between inclusion of contraception in the social health insurance and unplanned birth with a more causal interpretation than that which is often possible with population‐based studies [26]. By including an interaction term with the date of the last menstruation relative to January 1, 2004 in weeks, we investigated how much the odds of planned versus unplanned births changed right after the policy change, which reduces bias from macro‐level confounders. In addition, we repeated the analyses among participants with private insurance who did not experience a change in the coverage of contraception as a natural control population in order to evaluate the influence of potential unmeasured confounding factors. Also, some limitations should be considered. First, only pregnant participants were included in the Generation R cohort, which made it impossible to compare contraception rates before and after the policy change. Second, only pregnant people who intended to give birth and agreed on participation in a birth cohort study were included in this study. This means that pregnancies that ended in induced abortion and early miscarriages were not included. Moreover, those with unwanted pregnancies may have been less likely to participate, leading to an underestimation of the total number of unplanned pregnancies. National data did not show an increase in induced abortions after 2004, but we do not have data from Rotterdam specifically, so this may have influenced our findings [27]. Third, the study only included participants from the city of Rotterdam, which is the second largest city of the Netherlands [13]. Therefore, our findings may not be generalizable to less urban areas. Fourth, unplanned pregnancy was measured retrospectively in the first trimester using only one single item, while pregnancy planning or intention is much more complex and layered than one dichotomous variable. Moreover, the retrospective nature may have led to an underestimation of unplanned pregnancies [28, 29]. Fifth, no information was available on the reasons for not using contraception nor the choice of preferred methods. Therefore, we were unable to study whether the policy change may have led to limited choices, reduced autonomy, or financial burdens. Previous studies showed, for example, that long‐term methods with a high one‐time out‐of‐pocket spending—such as intrauterine devices—were more often chosen when they were provided free of charge [30, 31]. Finally, this study focused on women only. It is known that intention, attitude, and self‐efficacy regarding contraceptive use are higher among women than men [32]. In addition, gender minoritized populations face different barriers to contraception than cisgender women [33], suggesting that studying barriers to contraception in men and gender minoritized populations is also important.

In conclusion, the results of this study suggest that removing contraception from the social health insurance basic benefit package may have led to a small increase in unplanned births with a time lag of 6 months. Since we were unable to include pregnancies that resulted in miscarriage or induced abortions in our cohort, our results should be interpreted with caution. We recommend future studies to include all conceived pregnancies, as well as broaden their perspective to the impact on contraceptive choice to study health insurance policy changes in relation to reproductive autonomy.

## Conflicts of Interest

The authors declare no conflicts of interest.

## Supporting information


**Figure S1.** A–F: Percentage of different levels of socioeconomic variables on the *y*‐axis and date of conception on the *x*‐axis, plotted using the local regression method. The transparent area indicates the standard error.
A: Age categorized into 21–25 (orange), 26–30 (blue), 31–35 (green) and > 35 (pink) years.
B: Marital status categorized into married (orange), cohabiting (green) and single (pink).
C: Migration background categorized into yes (blue) and no (orange).
D: Educational level categorized into low (green), mid‐low (pink), mid‐high (blue) and high (red).
E: Household income categorized into low (green), medium (orange), and high (red).
F: Financial difficulties categorized into no (pink), some (orange) and great (blue).

**Table S1.** Odd ratios for planned vs. unplanned births using propensity score matching.
**Table S2.** Odd ratios for planned vs. unplanned births using propensity score matching, excluding participants with their last menstruation between January 1st 2004 and July 1st 2004.
**Table S3.** Odd ratios for planned vs. unplanned births using propensity score matching, excluding participants who additionally used condoms or other barrier methods in the previous five years.
**Table S4.** Odd ratios for planned vs. unplanned births using propensity score matching among participants with some or great financial difficulties or a low household income.
**Table S5.** Odd ratios for planned vs. unplanned births using propensity score matching among participants aged 21–25 years.
**Table S6.** Odd ratios for planned vs. unplanned births among the natural control population, the unadjusted model.
**Table S7.** Odd ratios for planned vs. unplanned births among the natural control population, the adjusted model.
**Table S8.** Odd ratios for planned vs. unplanned births using propensity score matching among the natural control population.
**Table S9.** Odd ratios for planned vs. unplanned births among the natural control population, excluding participants with their last menstruation between January 1st 2004 and July 1st 2004.
**Table S10.** Odd ratios for planned vs. unplanned births using propensity score matching among the natural control population, excluding participants with their last menstruation between January 1st 2004 and July 1st 2004.

## References

[1] E. Ketting , “Contraception and Fertility in The Netherlands,” International Family Planning Perspectives 8, no. 4 (1982): 141–147. 6680699

[2] H. de Graaf and C. Wijsen , “Seksuele Gezondheid in Nederland 2017,” 2017.

[3] E. Ketting and A. P. Visser , “Contraception in The Netherlands: The Low Abortion Rate Explained,” Patient Education and Counseling 23, no. 3 (1994): 161–171. 7971545 10.1016/0738-3991(94)90032-9

[4] H. de Graaf , “Monitor Seksuele Gezondheid 2023,” 2023.

[5] A. Baanders , “Bezuinigingen in de zorg: gevolgen voor chronisch zieken in het ziekenfonds,” 2004.

[6] E. van der Schee , Achterban coalitie en oppositie eensgezind in afwijzing maatregelen zorg (NIVEL, 2003).

[7] A. de Graaf , Geboorteregeling in 2008, ed. Bevolkingstrends (Centraal Bureau voor de Statistiek, 2009).

[8] M. Varkevisser , “Sustainability and Resilience in the Dutch Health System Partnership for Health System Sustainability and Resilience (PHSSR), London School of Economics and Political Science,” 2023.

[9] L. E. T. Swan , “Policy Impacts on Contraceptive Access in the United States: A Scoping Review,” Journal of Population Research 40, no. 1 (2023): 5.

[10] A. Gaffney and D. McCormick , “The Affordable Care Act: Implications for Health‐Care Equity,” Lancet 389, no. 10077 (2017): 1442–1452. 28402826 10.1016/S0140-6736(17)30786-9

[11] B. C. Wichmann , “Gratis anticonceptie is een zaak voor iedereen, hoger beroep 19 juni,” 2023, https://clara‐wichmann.nl/rechtszaken/gratis‐anticonceptie‐is‐een‐zaak‐voor‐iedereen‐hoger‐beroep‐19‐juni/.

[12] B. C. W. A. DeGoedeZaak , “Een zaak voor iedereen,” 2019, https://eenzaakvooriedereen.nl/.

[13] V. W. V. Jaddoe , J. P. Mackenbach , H. A. Moll , et al., “The Generation R Study: Design and Cohort Profile,” European Journal of Epidemiology 21, no. 6 (2006): 475–484. 16826450 10.1007/s10654-006-9022-0

[14] M. N. Kooijman , C. J. Kruithof , C. M. van Duijn , et al., “The Generation R Study: Design and Cohort Update 2017,” European Journal of Epidemiology 31, no. 12 (2016): 1243–1264. 28070760 10.1007/s10654-016-0224-9 PMC5233749

[15] M. Lipsitch , E. Tchetgen Tchetgen , and T. Cohen , “Negative Controls: A Tool for Detecting Confounding and Bias in Observational Studies,” Epidemiology 21, no. 3 (2010): 383–388. 20335814 10.1097/EDE.0b013e3181d61eeb PMC3053408

[16] S. T. Leatherdale , “Natural Experiment Methodology for Research: A Review of How Different Methods Can Support Real‐World Research,” International Journal of Social Research Methodology 22, no. 1 (2019): 19–35.

[17] C. A. Enthoven , H. El Marroun , M. E. Koopman‐Verhoeff , et al., “Clustering of Characteristics Associated With Unplanned Pregnancies: The Generation R Study,” BMC Public Health 22, no. 1 (2022): 1957. 36274127 10.1186/s12889-022-14342-y PMC9590126

[18] S. V. Buuren and K. Groothuis‐Oudshoorn , “Mice: Multivariate Imputation by Chained Equations in R,” Journal of Statistical Software 45 (2010): 1–68.

[19] P. C. Austin , I. R. White , D. S. Lee , and S. van Buuren , “Missing Data in Clinical Research: A Tutorial on Multiple Imputation,” Canadian Journal of Cardiology 37, no. 9 (2021): 1322–1331. 33276049 10.1016/j.cjca.2020.11.010 PMC8499698

[20] J. A. Labrecque and S. A. Swanson , “Using Counterfactual Worlds to Triangulate Evidence in the Real World,” Current Epidemiology Reports 11, no. 1 (2024): 44–53.

[21] F. Pishgar , N. Greifer , C. Leyrat , and E. Stuart , “MatchThem: Matching and Weighting After Multiple Imputation,” arXiv preprint arXiv:2009.11772, 2020.

[22] H. Grönqvist , Putting Teenagers on the Pill: The Consequences of Subsidized Contraception (Stockholm University, 2012).

[23] J. F. Peipert , T. Madden , J. E. Allsworth , and G. M. Secura , “Preventing Unintended Pregnancies by Providing no‐Cost Contraception,” Obstetrics & Gynecology 120, no. 6 (2012): 1291–1297. 23168752 10.1097/aog.0b013e318273eb56 PMC4000282

[24] B. G. Darney , R. L. Jacob , M. Hoopes , et al., “Evaluation of Medicaid Expansion Under the Affordable Care Act and Contraceptive Care in US Community Health Centers,” JAMA Network Open 3, no. 6 (2020): e206874. 32496568 10.1001/jamanetworkopen.2020.6874 PMC7273194

[25] T. Rau , M. Sarzosa , and S. S. Urzúa , The Children of the Missed Pill (National Bureau of Economic Research, 2017). 10.1016/j.jhealeco.2021.102496 PMC8496187 34399313

[26] M. A. Hernán and J. M. Robins , “Using Big Data to Emulate a Target Trial When a Randomized Trial Is Not Available,” American Journal of Epidemiology 183, no. 8 (2016): 758–764. 26994063 10.1093/aje/kwv254 PMC4832051

[27] G. van der Wal , Jaarrapportage 2005 van de Wet afbreking zwangerschap (Inspectie voor de gezondheidszorg, 2006).

[28] J. Santelli , R. Rochat , K. Hatfield‐Timajchy , et al., “The Measurement and Meaning of Unintended Pregnancy,” Perspectives on Sexual and Reproductive Health 35 (2003): 94–101. 12729139 10.1363/3509403

[29] C. H. Rocca , M. R. Wilson , M. Jeon , and D. G. Foster , “Stability of Retrospective Pregnancy Intention Reporting Among Women With Unwanted Pregnancies in the United States,” Maternal and Child Health Journal 23, no. 11 (2019): 1547–1555. 31236825 10.1007/s10995-019-02782-9 PMC6786959

[30] A. M. Gariepy , E. J. Simon , D. A. Patel , M. D. Creinin , and E. B. Schwarz , “The Impact of Out‐Of‐Pocket Expense on IUD Utilization Among Women With Private Insurance,” Contraception 84, no. 6 (2011): e39–e42. 22078204 10.1016/j.contraception.2011.07.002 PMC3217182

[31] L. E. Pace , S. B. Dusetzina , A. M. Fendrick , N. L. Keating , and V. K. Dalton , “The Impact of Out‐Of‐Pocket Costs on the Use of Intrauterine Contraception Among Women With Employer‐Sponsored Insurance,” Medical Care 51, no. 11 (2013): 959–963. 24036995 10.1097/MLR.0b013e3182a97b5d PMC6702955

[32] R.‐H. Wang , C.‐P. Cheng , and F.‐H. Chou , “A Causal Model of Contraceptive Intention and Its Gender Comparison Among Taiwanese Sexually Inexperienced Adolescents,” Journal of Clinical Nursing 17, no. 7 (2008): 930–939. 18321290 10.1111/j.1365-2702.2007.02088.x

[33] A. Bonnington , S. Dianat , J. Kerns , et al., “Society of Family Planning Clinical Recommendations: Contraceptive Counseling for Transgender and Gender Diverse People Who Were Female Sex Assigned at Birth,” Contraception 102, no. 2 (2020): 70–82. 32304766 10.1016/j.contraception.2020.04.001

