# Supplementary figures and images for: Health Insurance Coverage and Unplanned Births in Rotterdam, the Netherlands: A Natural Experiment in the Generation R Study
Source: Perspect Sex Reprod Health. 2025 Jun 21;57(3):293–300. doi: 10.1111/psrh.70020 (PMC12421080; doi:10.1111/psrh.70020)

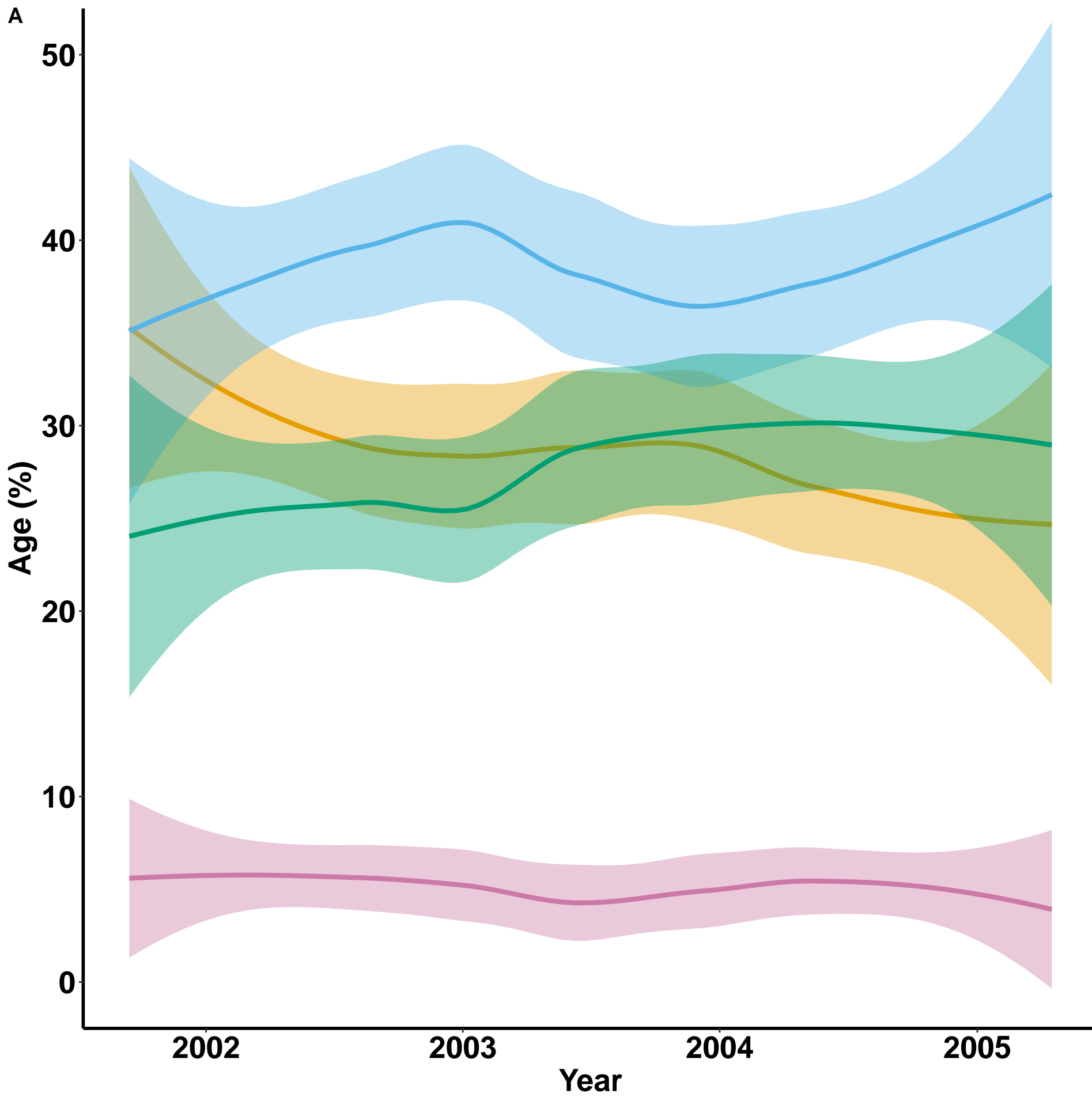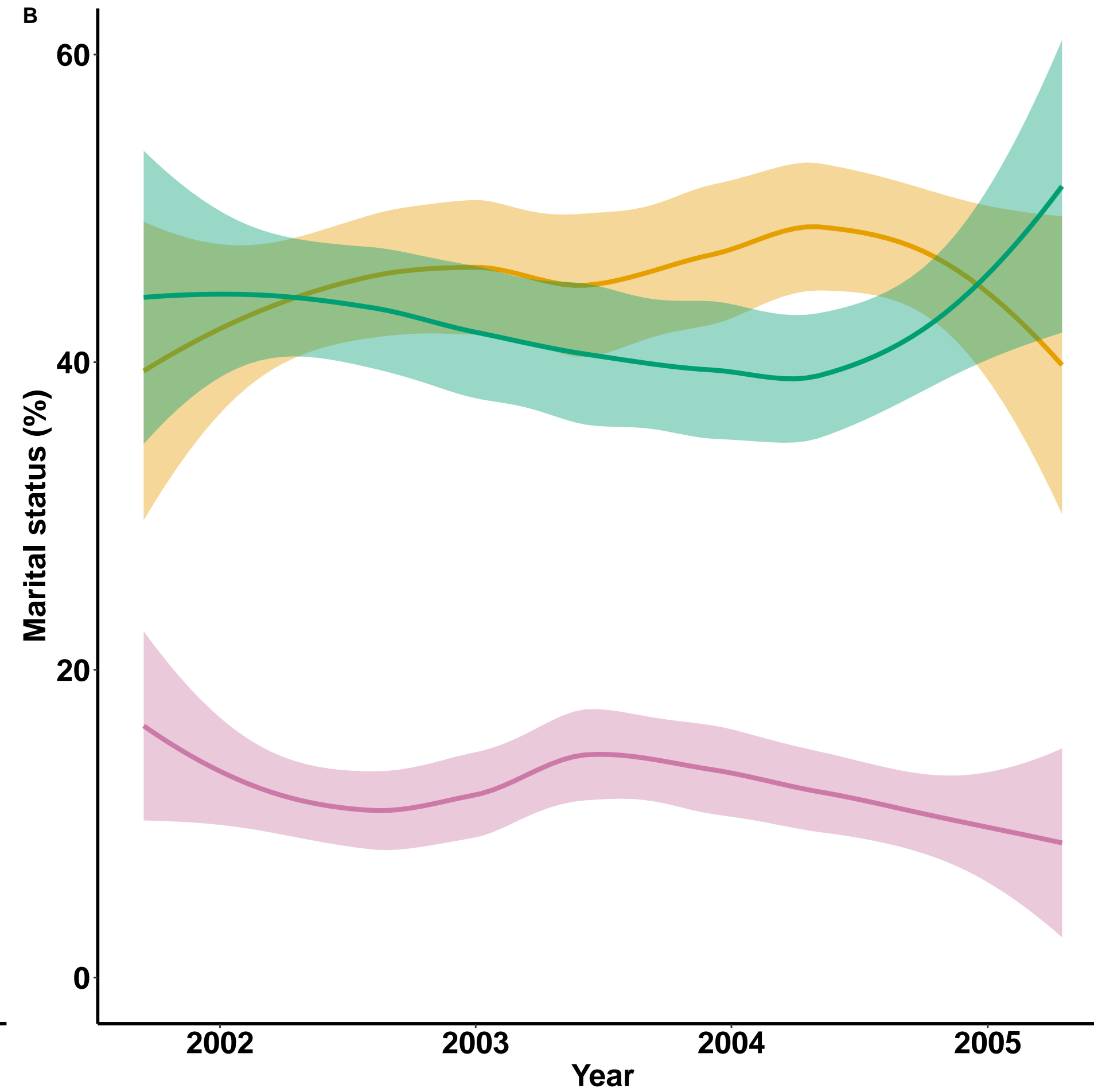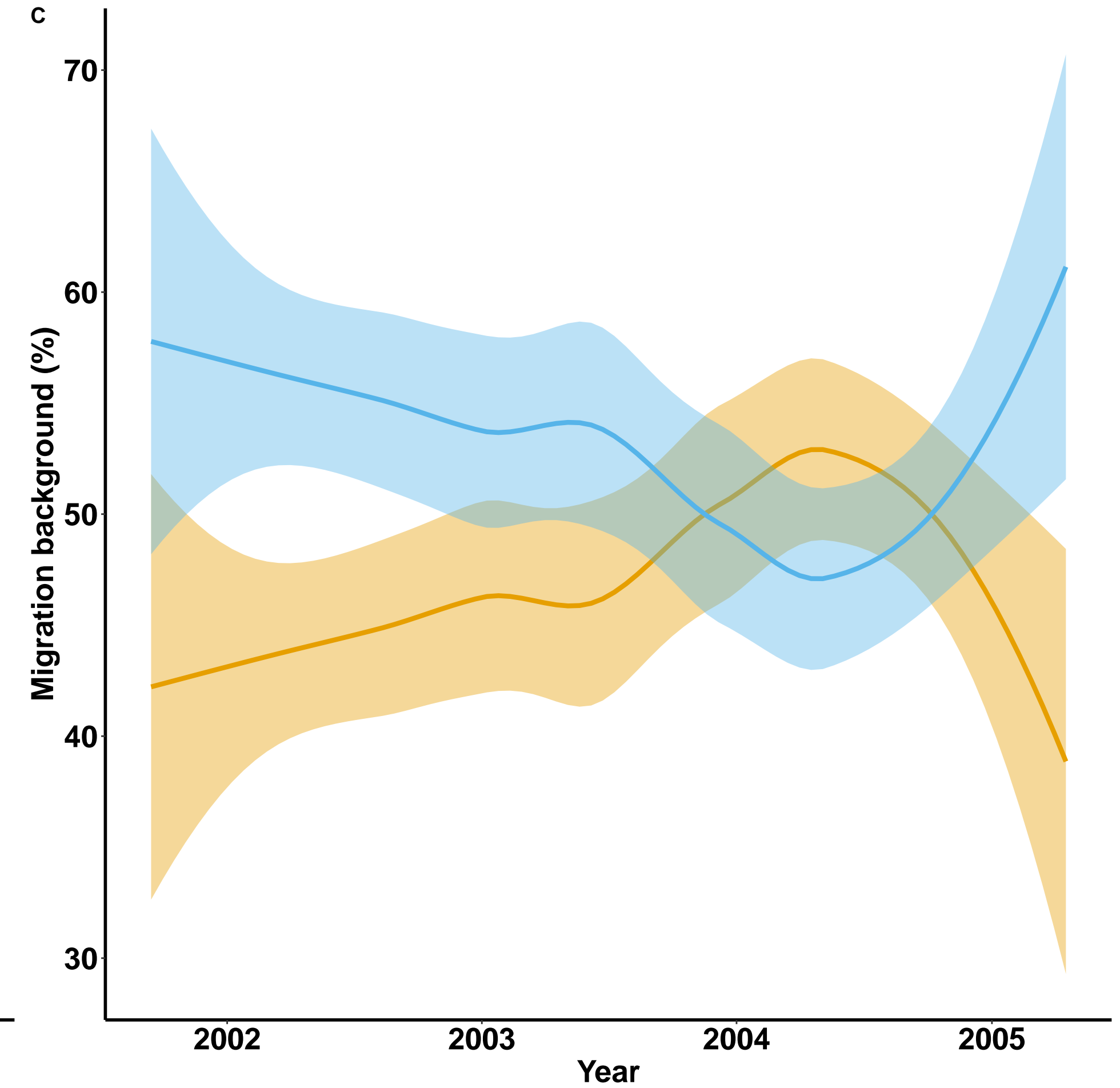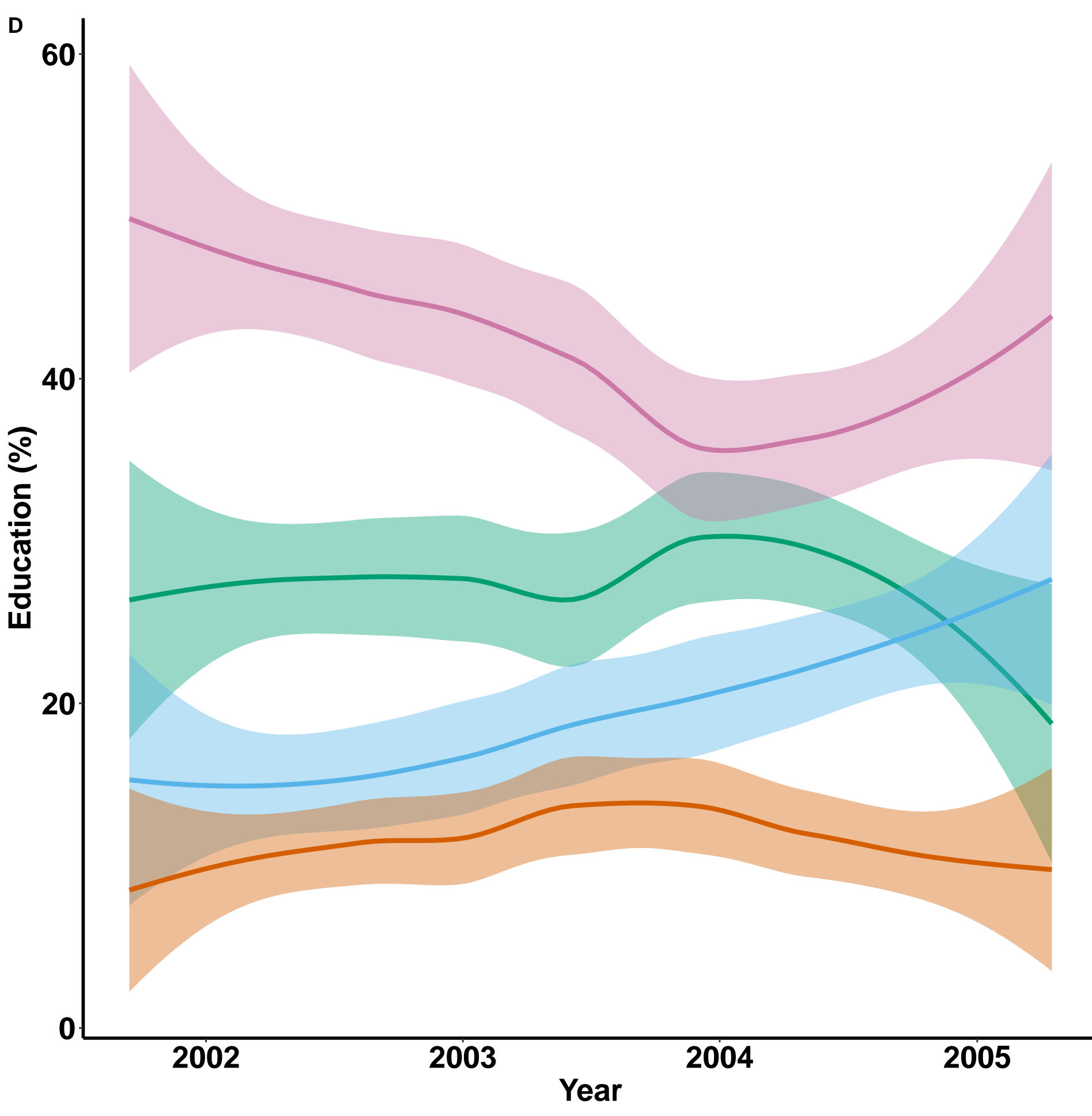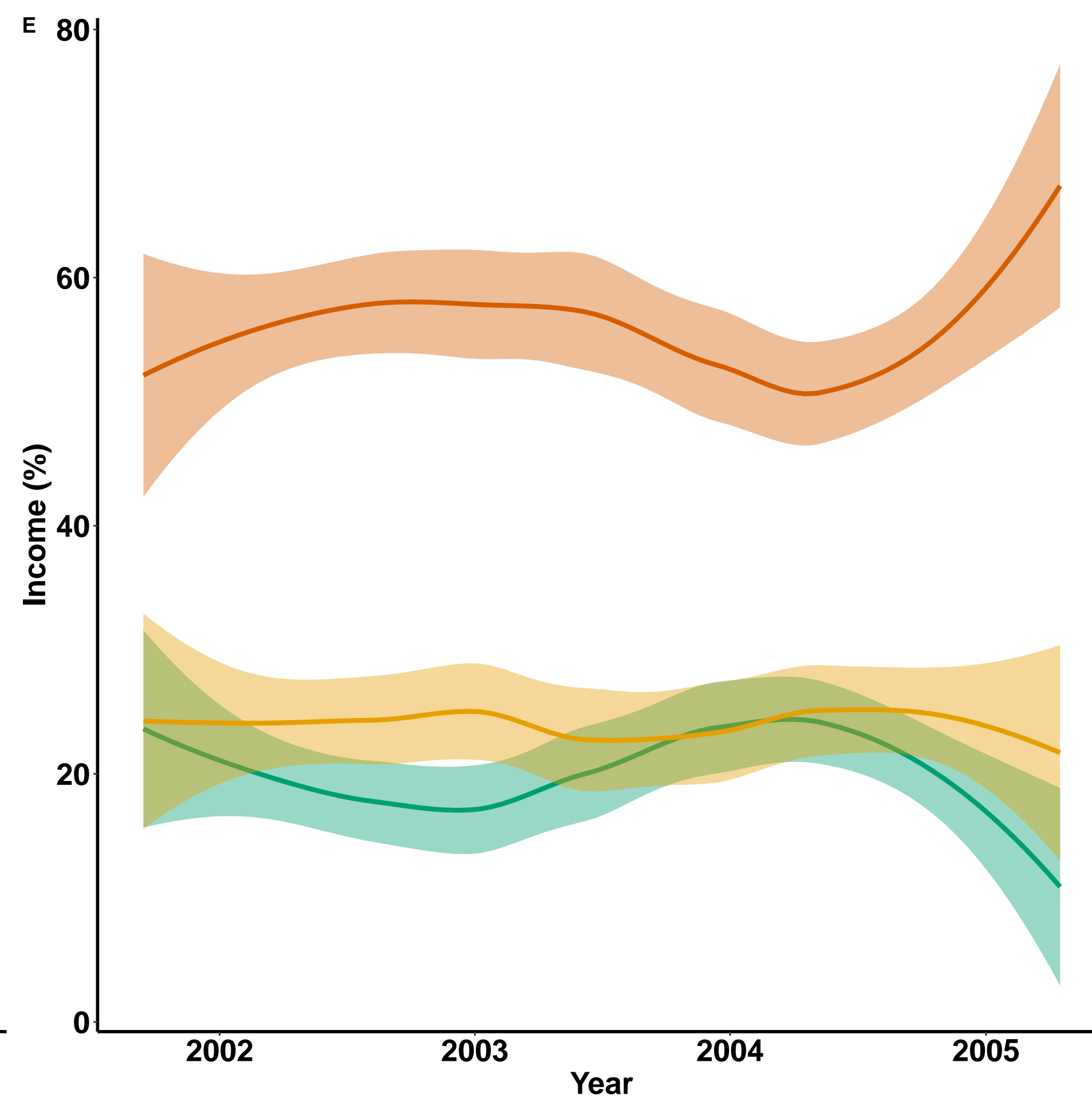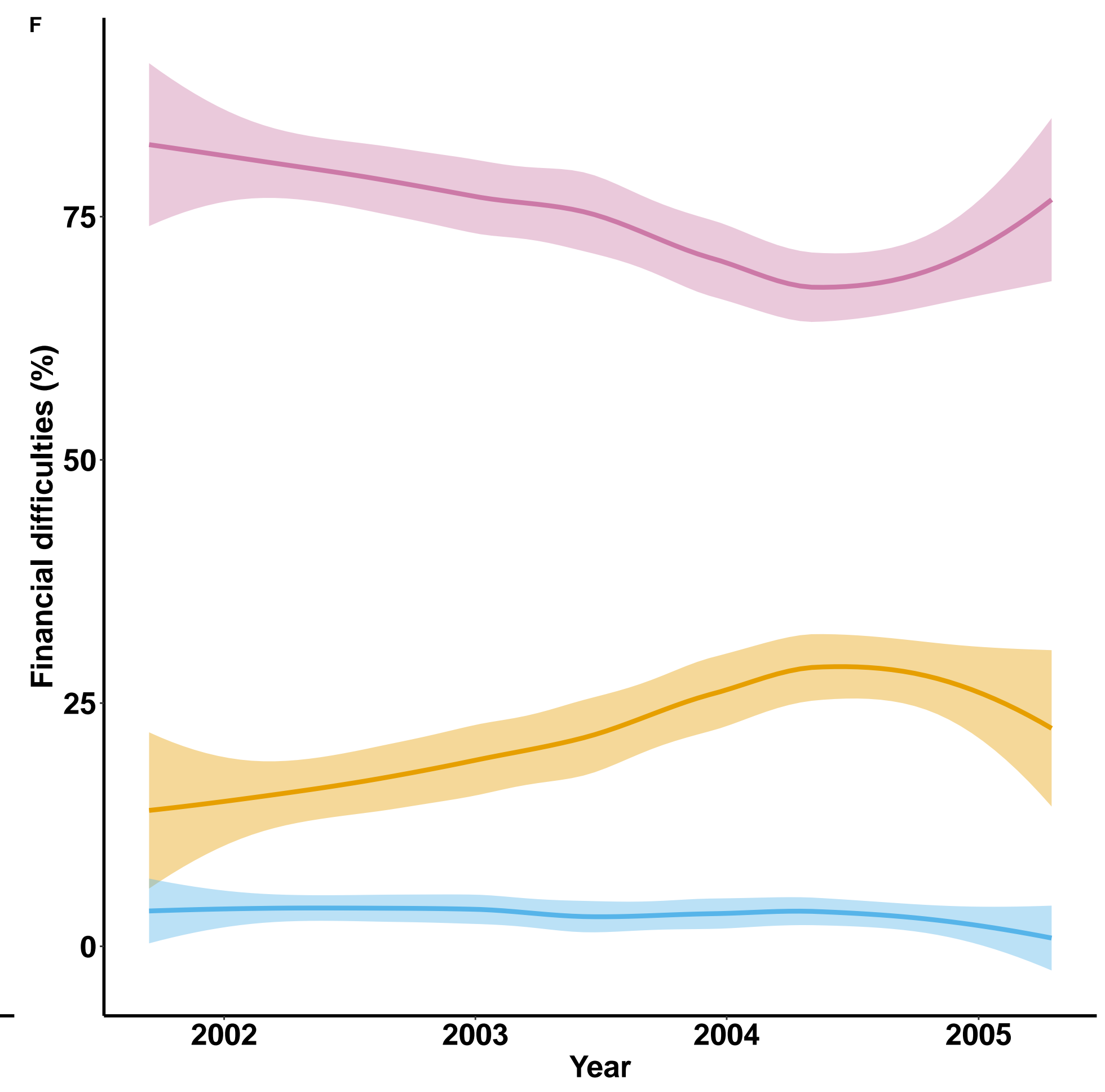

Supplement: Supplementary file 1 — Figure S1. A–F: Percentage of different levels of socioeconomic variables on the y‐axis and date of conception on the x‐axis, plotted using the local regression method. The transparent area indicates the standard error. A: Age categorized into 21–25 (orange), 26–30 (blue), 31–35 (green) and > 35 (pink) years. B: Marital status categorized into married (orange), cohabiting (green) and single (pink). C: Migration background categorized into yes (blue) and no (orange). D: Educational level categorized into low (green), mid‐low (pink), mid‐high (blue) and high (red). E: Household income categorized into low (green), medium (orange), and high (red). F: Financial difficulties categorized into no (pink), some (orange) and great (blue). [file PSRH-57-293-s001.pdf]
